# Why Neck Pain Patients Are Not Referred to Manual Therapy: A Qualitative Study among Dutch Primary Care Stakeholders

**DOI:** 10.1371/journal.pone.0157465

**Published:** 2016-06-16

**Authors:** Marije F. Dikkers, Marjan J. Westerman, Sidney M. Rubinstein, Maurits W. van Tulder, Johannes R. Anema

**Affiliations:** 1 Department of Health Sciences and EMGO Institute for Health and Care Research, Vrije Universiteit Amsterdam, De Boelelaan 1085, 1081 HV, Amsterdam, The Netherlands; 2 Department of Public and Occupational Health and EMGO Institute for Health and Care Research, VU University Medical Center, Van der Boechorststraat 7, 1081 BT, Amsterdam, Netherlands; Brown University, UNITED STATES

## Abstract

**Background:**

Treatment of neck pain with manual therapy demonstrated to be more effective and cost-effective than general practitioner (GP) care or physiotherapy in a high quality RCT in the Netherlands in 2002. However, referral to manual therapy for neck pain is still relatively low. This study aims to explore the barriers and facilitators affecting the implementation of manual therapy in neck pain management in primary care.

**Methods:**

An explorative study was conducted comprising semi-structured interviews with GPs (n = 13), physiotherapists (n = 10), manual therapists (n = 7) and their patients with neck pain (n = 27), and three focus groups with additional stakeholders (n = 10–12 per group). A thematic analysis approach was used.

**Results:**

Different barriers and facilitators for referral were found for patients, GPs and physiotherapists on the individual level, but also in the interaction between stakeholders and their context. Individual perceptions such as knowledge and beliefs about manual therapy for neck pain either impeded or facilitated referral. Fear for complications associated with cervical manipulation was an important barrier for patients as well as GPs. For GPs and physiotherapists it was important whether they perceived it was part of their professional role to refer for manual therapy. Existing relations formed referral behavior, and the trust in a particular practitioner was a recurrent theme among GPs and physiotherapist as well as patients. The contextual factor availability of manual therapy played a role for all stakeholders.

**Conclusions:**

Barriers and facilitators were found especially in individual perceptions on manual therapy for neck pain (e.g. knowledge and beliefs), the interaction between stakeholders (e.g. collaboration and trust) and the organizational context. Implementation strategies that focus on these different aspects seem to be likely to optimize referral rates and the use of manual therapy in primary care management of neck pain.

## Introduction

Neck pain is one of the leading causes of disability worldwide [1]. Besides disability, neck pain often results in substantial utilization of health care resources and absenteeism from work [2]. In the Netherlands, the total costs of neck pain are considerable and estimated at US $686 million per annum [2].

In the Netherlands, neck pain symptoms are commonly reported among general practice patients (incidence 23.1 per 1000 person-years) [3]. In most cases, there is no clear pathological basis for neck pain and the neck pain is labeled as non-specific [4]. In cases of non-specific neck pain, most frequently, the general practitioner (GP) advises the patient to ‘wait and see’ for an expected favorable natural course, usually supported with medication, or refers for physiotherapy [2], [5].

Physiotherapy may include active treatment, such as exercise therapies, and passive treatment, such as traction, massage and heat applications. Some physiotherapists may deliver manual therapy. Manual therapy can be described as “a specialized area of physiotherapy/physical therapy for the management of neuro-musculoskeletal conditions, based on clinical reasoning, using highly specific treatment approaches including manual techniques and therapeutic exercises”[6]. In the Netherlands, physiotherapists can specialize in manual therapy theory and techniques during a 3- to 4-year part-time course and register as manual therapists.

Numerous systematic reviews have demonstrated the effectiveness of manual therapy in patients with neck pain [7], [8], [9], [10], [11]. In addition, some studies have demonstrated the cost-effectiveness of manual therapy for neck pain [12], [13]. A relatively large, well-conducted randomized controlled trial (RCT) with an economic evaluation alongside in the Netherlands in patients with non-specific neck pain demonstrated that manual therapy was less costly and more effective compared to physiotherapy or GP care [13], [14].

Despite the documented benefits of manual therapy for patients with neck pain, GP referral to manual therapy in patients with neck pain is very low. According to a Dutch national GP registry, from 2007 to 2011, among the one fifth of the patients with neck pain referred by the GP, 70% were referred to physiotherapy but only 8% to manual therapy [15]. These rates suggest that a very small proportion of the patients with neck pain are directed towards manual therapy and managed in the most cost-effective manner.

There may be many reasons for the gap between this scientific evidence on neck pain management and its use in daily practice. For example, reasons for non-adherence to scientific recommendations may be related to patients’ experiences in the past, GPs’ interpretations of the patients’ preferences, and to GPs’ delegation of the decision about therapy to the therapists, thinking it to be beyond their competency [16]. However, no research has addressed the low referral rate to manual therapy in the primary care management of neck pain. Bridging this evidence-practice gap requires an in-depth understanding of the barriers and facilitators to referring patients with neck pain for manual therapy. Factors that impede or facilitate the implementation of evidence in patient care are found at various levels, and include factors related to the innovation, the professionals and patients involved, and the social, organizational, economic and political context [17], [18]. This framework can be used to identify barriers and facilitators, and so to tailor interventions to facilitate desired change [17].

The aim of the present study was to investigate the barriers and facilitators to manual therapy referral in neck pain management in primary care. Since GPs, patients and therapists are all important stakeholders in the primary care decision chain with regard to the use of manual therapy treatment [16], [19], we explored the barriers and facilitators among these different stakeholders. The results of this study help to tailor interventions to facilitate change, thus improving quality of care for this patient group and cost savings for society.

## Methods

### Study design

A qualitative explorative study was performed using semi-structured interviews and focus group meetings. The interviews were conducted to collect rich and in-depth information about the possible barriers and facilitators to manual therapy referral. The focus groups were conducted to deepen understanding of the findings from the interviews and to explore possible strategies to address the identified barriers and facilitators. Tables 1 and 2 show more details on the participants in the interviews and focus groups.

**Table 1:** Participant characteristics interviews (n = 57).

** Patients**				
** **	**recruited via GP (n = 10)**	**recruited via PT (n = 10)**	**recruited via MT (n = 7)**	**Total (n = 27)**
**Gender** male/female	4/6	2/8	3/4	9/18
**Age** years, average (range)	49 (28–66)	49 (39–69)	47 (25–65)	48 (25–69)
**Education** high/intermediate/basic	1/5/4	5/1/4	2/3/2	8/9/10
**Entry** referral/self-referral	N.A.	3/7	2/5	N.A.
**Practitioners**				
	**GP (n = 13)** ^	**PT (n = 10)**	**MT (n = 7)**	**Total (n = 30)**
**Gender** male/female	6/7	6/4	4/3	
**Age** years, average (range)	48 (34–58)	39 (23–57)	44 (29–57)	
**Years of experience**, average (range)				
as a GP	15 (4–32)			
as a PT		14 (0.5–29)	18 (5–33)	
as a MT			10 (2–25)	
**Type of practice** solo/group/multidisciplinary*	2/7/4	2/7**/1	1/5/1	
**Location of practice** city/other (suburban, rural)	9/4	7/3	2/5	

GP: General Practitioner; PT: Physiotherapist; MT: Manual therapist

* practice with one practitioner / practice with several GPs or PTs/MTs) / center with various disciplines (e.g. GPs and therapists)

** 5 group practices with MT and 2 without

^ 3 GPs were interviewed without an included patient, to ensure data saturation before the end of the data collection

**Table 2:** Participant characteristics focus groups (n = 33).

	Focus group 1 (n = 12)	Focus group 2 (n = 10)	Focus group 3 (n = 11)
GP	4		4
PT	3		3
MT	4		4
Research coordinator Hoving trial	1		
Representative health care insurer (Platform Health Care Insurers Paramedical Care)		1	
Representative Dutch Health Care Insurance Board (CVZ)		1	
Representative Royal Dutch Association for Physical Therapy (KNGF)		1	
Representative Dutch Association for Manual Therapy (NVMT)		1	
Representative Netherlands Society of Occupational Medicine (NVAB)		1	
Representative Dutch Association for Back Pain Patients (NVVR)		1	
Representative GP/guideline maker for Dutch College of GPs (NHG)		3	
Representative guideline maker for Dutch Association of PT/MT (KNGF)		1	

GP: General Practitioner; PT: Physiotherapist; MT: Manual therapist

### Interviews among practitioners and their patients

During the period of July 2012 to March 2013, we conducted 57 semi-structured interviews with GPs, physiotherapists, manual therapists and their patients. We recruited practitioners, seeking variety of practice type (solo/group/multidisciplinary), location of practice (city, suburban, rural), years of practice, age and gender. Members of a GP and a physical therapist network at the Vrije Universiteit were invited by e-mail, and a call for practitioners to participate was placed on the website of a regional primary care agency. Follow-up telephone calls were made with selected members of the networks to invite practitioners with specific characteristics not yet included in the sample (e.g. considering diversity in years of practice).

Each practitioner was asked to recruit a patient at his/her practice. Inclusion criteria for patients were pain and/or stiffness in the neck for at least two weeks, and age between 18 and 70 years. Exclusion criteria were specific underlying pathology, previous surgery for neck complaints and insufficient mastery of the Dutch language [20]. If the first eligible patient refused to participate, the practitioner was asked to invite the subsequent patient to participate. If the patient gave consent, both patient and practitioner were interviewed. The interviews were scheduled shortly after patient inclusion. This allowed the reasons and motives concerning the recent patient visit to be explored with both parties.

Topic lists were used and revised during the study to include issues that emerged as important in early interviews. A distinct topic list was used for each target group. In order to understand the context and to give participants the opportunity to express themselves in their own words, all participants were asked to tell the interviewer more about the recent consultation for neck pain. Patients were asked to talk about their neck pain and were asked about the care they received, their views on manual therapy and visiting the manual therapist to treat their neck pain. GPs, physiotherapists and manual therapist were asked to talk about their considerations in the care provided to the patient and were allowed to use their notes in the patient file as reminders (e.g. reason for consultation, diagnosis, treatment plan and motivation). In addition, practitioners were asked about their general management of patients with neck pain and their use of manual therapy in particular (e.g. for GPs and physiotherapists: do they sometimes advise patients to visit a manual therapist?). During the interview, the interviewer directed the conversation towards manual therapy for non-specific neck pain. To encourage the participants to talk freely about manual therapy for neck pain, they were initially not informed about the precise focus of the study.

The interviews were held by MFD, the principal investigator, a trained interviewer with a master’s degree in health sciences. Interviews were conducted at time and locations convenient for the participants (the patients’ homes or offices, or in the practitioners’ offices), and generally took place within one week following patient inclusion (range 1–18 days). The practitioner interviews lasted, on average, one hour (range 45–75 minutes) and most patient interviews approximately 30 minutes (range 20–60 minutes). Field notes were written up directly after each interview and included thoughts and comments about the participants’ perspectives. Interviews were voice-recorded and transcribed verbatim. Interviews were scheduled until data saturation was reached.

### Focus groups with various stakeholders

Three focus group meetings with various stakeholders (10 to 12 participants per group) were conducted. In January 2013, a focus group was conducted in Zoetermeer with practitioners who had participated in the Dutch (cost-)effectiveness study in patients with non-specific neck pain [20]. This enabled us to investigate whether and how these practitioners, who were familiar with the trial, used the trial outcomes in their current practice. To gain further understanding from a policy perspective, a focus group was conducted with policy makers from different parties involved in the implementation of manual therapy (e.g. guideline makers, health insurance advisors, and practitioner and patient associations) at the Vrije Universiteit Amsterdam in February 2013. To assay the provisionally identified barriers and facilitators, a focus group with additional professionals (GPs, PTs, MTs) was conducted at the regional meeting office of their care group in March 2013.

Recruitment of the focus group participants took place via different routes. Previous participating practitioners in the Dutch trial were recruited via the coordinator of that study and received a posted invitation. Policy makers were recruited by means of an e-mailed invitation to the selected associations and to appropriate professionals in our own network. Additional practitioners (GPs, PTs, MTs) were recruited in a new region, via a regional care group and our own network, and received a mailed invitation. Follow-up telephone calls were made to invite particular professionals with specific characteristics not yet included in the sample (e.g. considering the professional background).

During each focus group, a topic guide was used. We explained the background of the study. A brief overview of the provisionally identified barriers and facilitators was presented and discussed (i.e., did they recognize them; were any missing). Next, participants were asked what they considered to be the most important barriers and facilitators. The group discussion enabled further exploration of how the different themes and categories interacted with one another. Subsequently, participants were asked for possible solutions to improve implementation, taking these barriers and facilitators into account. We used this format to stimulate discussion about factors that could be changed, and to explore potential strategies to address these. For each focus group, the subtopics to be discussed were adjusted to the shared background of the participants and the provisional findings.

All focus groups lasted two hours, and were voice-recorded and transcribed verbatim. The sessions were moderated by the principal investigator (MFD). A second member of the research team acted as observer and co-moderator (SMR or MJW), and an assistant made observations and took field notes.

### Analysis

Data were collected and analyzed concurrently, allowing emerging themes to be incorporated and explored in subsequent interviews. Thematic content approach was used in the analysis of the transcripts [21]. We explored patients’ and practitioners’ behavior, views and motives concerning manual therapy referral for neck pain. We looked for themes at different levels for implementation in patient care [17], [22], considering factors related to manual therapy itself, the patient, the individual professional, and the context (social, organizational, political and economic factors).

Throughout the analysis process, memos were written, including notes about the identified themes and the relationships between these themes, and provisional findings were critically discussed in research team meetings with all authors. To facilitate coding, organizing, and selecting data from transcripts, MaxQDA 10 was used.

All transcripts were read closely and analyzed. A summary of each interview was made based on the first analysis and the field notes. The first interviews in each target group were independently coded by two investigators (MFD and SED). The initial barriers, facilitators and themes identified by the two investigators were discussed in meetings with a third investigator (MJW), who reviewed sample transcripts, the coding scheme, and analytical decisions. After several meetings of the investigators, focusing on understanding the collected data and correct interpretation, an initial thematic map was developed. Within the themes we distinguished barriers and facilitators. Next, the data was coded either by MFD or SED, and iterative discussion between the researchers took place. In this phase, we reviewed themes for coherence, refined themes, identified new themes, recoded some data extracts, and sorted and collated the barriers and facilitators according to overarching themes. The two researchers discussed the refined and new themes until consensus was reached. If controversy remained, the other research team members (MJW, SMR, JRA, MWT) were consulted to come to a decision.

### Ethical considerations

The medical ethical committee of VU University Medical Center Amsterdam gave approval for the study and declared that the study does not fall within the scope of the Medical Research Involving Human Subject Act (ref. number 2012/170). Written informed consent was obtained from all participants at each interview.

## Results

### Different pathways

**Fig 1:**
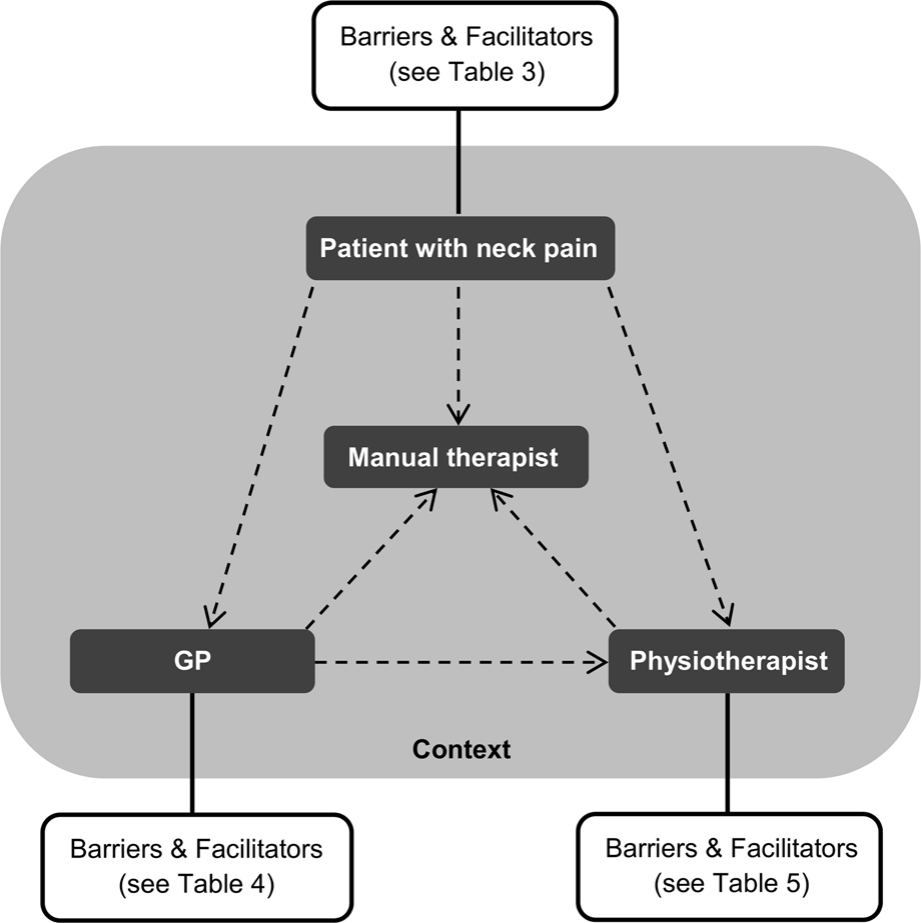
Model with pathways to the manual therapist for a patient with neck pain. In Fig 1 the arrows illustrate the different possible pathways to the manual therapist for a patient with neck pain: via self-referral, via the GP, and via the physiotherapist. Different barriers and facilitators for referral were found for patients, GPs, and physiotherapists. Factors were found on the individual level, but also in the interaction between stakeholders, and their context (Tables 3–5).

All patients told that they searched for care because their neck complaints lasted too long or the complaints became more severe. They wanted to know what caused their complaints, and to receive advice or a solution. Patients choose to walk different pathways within primary care, and their interaction with a GP or physiotherapist could influence the continuation of their pathway (Fig 1).

One pathway is direct access to manual therapy. Some patients went to the manual therapist on their own initiative. They had good experiences with manual therapy for other complaints, heard positive stories about a manual therapist from other people, or read information about manual therapy on the Internet. However, most patients did not visit the manual therapist directly.

A second pathway is via the physiotherapist. Some patients went to the physiotherapist first. They were not familiar with manual therapy or chose knowingly to visit a physiotherapist. While applying for a consultation at the practice, a few were advised to be treated by a manual therapist. However, most were directly scheduled to be seen by a physiotherapist. Nearly all physiotherapists said they usually started with their own treatment, but sometimes advised patients with neck pain to visit a manual therapist. Mainly if they found an arthrogenic restricted movement they could not treat themselves or for a second opinion occasionally.

The third pathway starts with visiting the GP. Some patients went to their GP first, because they wondered whether the cause was ‘something serious’ and what could help to reduce the complaints. A single one first went to the GP specifically for a referral to save extra costs for a physiotherapy screening after self-referral.

Most GPs said they regularly referred patients with neck pain to physiotherapy, but did not or rarely refer to manual therapy. GPs told they only referred to manual therapy in exceptional cases such as a ‘blockade’ of the joints. The few GPs who did routinely refer patients with neck pain to manual therapy, said they started this after they participated in and gained knowledge about the results of the Hoving trial [20] or after a personal explanation from a local manual therapist about manual therapy and its value in neck pain management.

### Barriers and facilitators

Different barriers and facilitators were found for patients with neck pain to be referred to manual therapy among the different stakeholders (Fig 1). The different barriers and facilitators found for patients, GPs and physiotherapists are presented in Tables 3–5, respectively. Factors were found on the individual level, such as a GP’s knowledge, but also in the interaction between stakeholders, and their context (e.g. policies in the physiotherapy practice). Within this field we found overarching themes. These themes are described more in-depth below, and are organized into three main categories: I) view on manual therapy for neck pain, II) collaboration and trust, and III) organizational context.

**Table 3:** Barriers (b) and facilitators (f) for patients with neck pain to visit the manual therapist.

Factor	Explanation of factor (barrier and/or facilitator)
**View on manual therapy for neck pain**	
Awareness	P is aware (**f**) / not aware (**b**) of the option of manual therapy for neck pain
Beliefs and attitudes	P perceives manual therapist as physiotherapist with expertise in the neck region (**f**)
	P has a fear for manipulation and its possible complications and aftereffects (**b**)
Preferences	P perceives manual therapy does **(f**) / does not (**b**) match with his or her preferences for a particular approach (e.g. idea of ‘harder’/‘firmer’ therapy than physiotherapy, active versus passive approach)
**Care experiences**	
Experiences with manual therapy	P has a positive (**f**) / negative (**b**) previous experience with manual therapy
Experiences with other care	P tried other options (e.g. pain medication, physiotherapy) without success (**f**)
**Resources**	
Network	People from P’s social or care network advise (**f**) or discourage (**b**) manual therapy
Media	P takes in positive (**f**) / negative (**b**) information about manual therapy in the media (e.g. on the Internet)
Financial resources	P receives a reimbursement from the health care insurer for the treatment (**f**)
Physical environment	P visits a practice wherein a manual therapist is not available (on short notice) (**b**)

P: Patient

**Table 4:** Barriers (b) and facilitators (f) for GPs to refer patients with neck pain to the manual therapist.

Factor	Explanation of factor (barrier and/or facilitator)
**View on manual therapy for neck pain**	
Knowledge	GP has knowledge (**f**) / lack of knowledge (**b**) about the indication for and nature of manual therapy in neck pain management
Beliefs and attitudes	GP perceives manual therapist as a physiotherapist with extra education in amongst others the neck region/spine (**f**)
	GP has a positive (**f**) / negative (**b**) attitude towards the evidence about the effectiveness of manual therapy for neck pain
	GP fears risk of/serious complications associated with manipulation (**b**)
	GP perceives manual therapy as care that does not provide a long-term solution (**b**)
**Collaboration and trust**	
Negotiation with the patient	GP perceives manual therapy does (**f**) or does not (**b**) match with the characteristics of the individual patient (e.g. preferences), estimation or attested after debate
Role orientation	GP perceives it does (**f**) / does not (**b**) belong to his or her professional role to decide between an indication for manual therapy or physiotherapy (e.g. beyond competency)
Work relations with therapists	GP has trust (**f**) / lack of trust (**b**) in method of treatment of the particular manual therapist
Insight in local expertise	GP has insight (**f**) / a lack of insight (**b**) in the expertise and approach of the manual therapists in his or her local network (e.g. who does what)
**Organizational context**	
Availability of manual therapy	GP encounters a lack of availability of manual therapy in direct work environment (**b**)

**Table 5:** Barriers (b) and facilitators (f) for physiotherapists to advise patients with neck pain to visit the manual therapist.

Factor	Explanation of factor (barrier and/or facilitator)
**View on manual therapy for neck pain**	
Perceptions	PT perceives manual therapists to have additional diagnostic and technical skills concerning the neck region/spine (e.g. manual therapy perceived to be suitable for arthrogenic movement restrictions and a 2^nd^ opinion) (**f**)
	PT doubts whether manual therapy has a possible added value above physiotherapy for particular patients with neck complaints (**b**)
**Collaboration and trust**	
Role orientation	PT is tended to adopt the role of ‘the Practitioner’ (treats until maximum benefit is reached with own treatment) (**b**) / the role of ‘the Care Manager’ (directing patient towards optimal care, potentially treatment by another care provider) (**f**)
Work relations	PT has trust (**f**) / lack of trust (**b**) in method of treatment of the particular manual therapist, inside or outside the practice
**Organizational context**	
Availability of manual therapy	Lack of availability/capacity of manual therapist(s) in practice (**b**)
Practice policy	Practice is working according to the ‘specialist model’ (patients are led to the therapist with the particular expertise) (**f**)
Culture of consultation	Approachable culture of consultation between the therapists in the practice (e.g. aware of and using each other’s strengths) (**f**)
Financial interest	Financial interest of the practitioner/organization is at stake (**b**)
Regulations	Practice is not allowed to declare manual therapy for a patient with a physiotherapy referral (**b**)

PT: Physiotherapist

### View on manual therapy for neck pain

#### Manual therapy: what is it?

Patients and professionals had different views on what manual therapy is. Patients and professionals spoke about manual therapy in a narrow sense, as they were referring to spinal manipulation and particular passive mobilization techniques, or in a broader sense. In the latter case, they referred to specific clinical reasoning and specific treatment supplementary to the field of general physiotherapy. Those with a broader view seemed to consider manual therapy for neck pain more often. For example, all GPs who regularly referred patients with neck pain to manual therapy brought up the specialized training manual therapists followed in diagnostics and treatment after their physiotherapy education.

Both physiotherapists and manual therapists pointed out that the transition between the domain of manual therapy and physiotherapy is a grey area. A representative of the Dutch Association for Manual Therapy said:

*“I think a problem lies in the division between physiotherapy and manual therapy*. *Where does physiotherapy end and manual therapy begin*? *I think for primary and secondary care stakeholders*, *the distinction between manual therapy and physiotherapy is difficult to make because there is a big overlap”*.

GPs acknowledged that the difference between manual therapy and physiotherapy was not entirely clear for them. Patients encountered the same indistinctness, and even some patients treated by a manual therapist were not aware they received manual therapy. Illustratively, one patient remarked:

*"I think all physiotherapists are manual therapists*, *aren't they*? *[…] At first*, *you just go to physiotherapy” (female*, *age 52*, *receiving manual therapy after self-referral)*.

Furthermore, patients, GPs and therapists pointed out that they did not perceive manual therapy as one fixed intervention, since it is given by a particular therapist with his or her characteristics, approach and use of particular techniques, which can be quite different depending on training and personal style.

#### Manipulation and possible complications

Several patients, GPs and physiotherapists associated manual therapy with manipulation of the joints (often called “cracking”). A few patients expressed their fear for manipulation of their neck and possible complications and aftereffects, and told this impeded them from visiting a manual therapist. They preferred to receive physiotherapy and perceived manual therapy only as an end-of-the-line option. One of these patients, who had a previous experience with manual therapy for another complaint, described:

*“I think it is an intense treatment […] You don’t have control over the situation at all*. *So when the treatment is too hard*, *it is too late to respond*. *Well*, *that is what happened to me*, *and you suffer the consequences for a very long time*. *I don’t like that*. *If nothing else works*, *OK*, *then you undergo it*.*” (female*, *age 44*, *receiving physiotherapy after self-referral)*.

Therapists and GPs recognized the fear for manipulation and (anticipated) unpleasant experiences among patients. Some GPs and also a physiotherapist said they were fearful about complications associated with cervical manipulation themselves, and that this impeded them to refer for manual therapy.

#### The appropriateness of manual therapy

The majority of the GPs and physiotherapists said to be unfamiliar with the scientific knowledge on the (cost-)effectiveness of manual therapy compared to physiotherapy and GP care for neck pain. A few GPs expressed they assumed manual therapy and physiotherapy would be less cost-effective, considering the direct cost of the treatment. Some GPs and physiotherapists said that the scientific evidence about manual therapy for the treatment of neck pain was not (or would not be) conclusive for them. For example, one GP argued that the study results of the Hoving trial were not relevant for the individual patient sitting in front him. Others questioned whether manual therapy would have an added value above physiotherapy or GP care. Professionals and patients thought there was not one treatment that is clearly better than others.

GPs and therapists emphasized that in deciding what is relevant for the individual patient, they especially considered characteristics such as the origin of the complaints, which can be myogenic and also stress or posture related. Some doubted whether treatment by a manual therapist could have an added value in such cases, since manual therapists are specialized in improving the function of the joints. For example, a physiotherapist said that manual therapists have a specific focus: “*those joints have to move*”, and that this focus on the joints was not adequate for any patient, such as the patient he included for this study:

*“There are no joint problems*. *There is a muscle problem*. *I think a manual therapist would have focused too much on that rotation restriction and wouldn’t have paid enough attention to the muscle part” (male*, *age 24*, *solo practice)*.

The perception of manual therapy as an entirely or mainly passive treatment was also an issue. For example, some GPs and also a patient said they perceived manual therapy as a more passive treatment than physiotherapy, and incompatible with an active or self-management approach and long-term outcomes. A GP stated:

*“I don’t refer patients to the manual therapist because I am not convinced of the usefulness of cracking and straightening things*. *I think that it gives relief*, *I hear that from people*, *but I don’t believe that it solves the problem” (female*, *age 37*, *group practice)*.

Conversely, some patients said they have confidence especially in the passive part of the therapy. Passive treatment gave them the idea that the right spots were well addressed by the trained therapist, while patients doubted whether they performed their home exercises in the right way by themselves. However, some physiotherapists believed that such patients and manual therapy itself merely focus on passive treatment and thus miss an important focus that is incorporated in their own therapy, namely prevention of recurrence.

### Collaboration and trust

#### Collaboration and trust: Patients’ perspective

Table 3 reports the barriers and facilitators that patients experienced. Patients decided to which practitioner or practice they went on the basis of trust. They went to a therapist they already had positive experiences with for the treatment of another musculoskeletal complaint, either a manual therapist or a physiotherapist. Others used the recommendations from their family or friends to decide. Some proactively used the Internet to find a practice or therapist that felt right for them. For example, the fact that a practice had multiple specializations or the information that a manual therapist had expertise in treating particular neck complaints appealed to them. Those who visited a practice with multiple therapists often followed the suggestion of the receptionist about which therapist to see. Others told they fully trusted the advice from their GP.

*“There also are people who go googling*: *What is a manual therapist*? *What is the difference*? *What does he add [to physiotherapy or GP care]*? *Well*, *that is not really how I am*. *I trust the people who I visit*. *We already visit the GP as long as I live here and we have a pleasant contact with that man*. *So I just assume that man recommends a therapist from his vision with whom he has a good experience” (male*, *age 65*, *receiving manual therapy after GP referral)*.

The interviews with patients and GPs showed that sometimes patients were advised by their GP to go to a particular therapist or practice and sometimes they only were referred to “physiotherapy”.

#### Collaboration and trust: GPs’ perspective

Table 4 presents the barriers and facilitators that GPs reported. GPs said they tailored their plan to, and in consultation with, the individual patient with his or her characteristics such as the patient’s preferences, experiences and personality. They pointed out that negotiation and debate with the patient were part of the decision making process, and that it could be difficult to convince a patient that he or she could benefit from manual therapy. GPs exemplified that some patients have a strong desire for medication as they have no time or money for therapy, some are eager to work on it themselves, and others believe only diagnostic imaging and operations can help them. Some GPs said only to refer for manual therapy if patients mooted they had positive experiences with manual therapy or had no confidence in physiotherapy.

Most GPs said they usually delegated the decision whether there was an indication for manual therapy to the physiotherapists. Their lack of knowledge about indications and contra-indications was mentioned as a barrier to refer specifically for manual therapy. Some GPs thought that such decisions belonged to the field of physiotherapy and was beyond their competency, as one of them said:

*“I have the impression that physiotherapists are better able to assess that than I am*. *[…] We [GPs] feel responsible for the diagnostics*, *and determining the treatment trajectory the patient enters*. *And if in a next step in the treatment trajectory*, *manual therapy seems to be an option*, *then that is fine*. *However*, *we [GPs] like to leave it to the physiotherapists*. *[…] We think it does not belong to our task to do that*. *[…] We say that is typically something for the physiotherapist” (male*, *age 51*, *group practice)*.

However, other GPs wanted to make a distinction between an indication for manual therapy or physiotherapy themselves, so that the patient would be at the right place immediately.

GPs told that insight in the expertise of therapists (‘who does what’) helps them to be more specific in their referrals. This insight varied from knowing what kind of specializations therapists have to familiarity with the approach and personality of a particular therapist. For example, one GP told she even tried to match the personality of the patient with the personal approach of the manual therapist.

For some GPs trust in the treatment by and collaboration with particular therapists was leading. They told they preferred to refer patients to therapists of whom they knew were very competent and to practices with various specializations whereof they knew there was a good mutual collaboration between therapists. By doing so, they trusted that a physiotherapist would consult a manual therapist if necessary, as a GP said:

*“I would never refer a patient for just ‘manual therapy treatment’*. *Actually*, *I only want to refer patients to therapists I know well*, *and of whom I know they don’t go too far in a treatment*. *And the people who do manual therapy here*, *within this team [GP refers to PT/MT practice in the same building]*, *of them I know that they are not incompetent or don’t cause damage and don’t go on too long*, *and that they do it if it is necessary*. *And I leave that to them entirely*. *I have a very good and peaceful feeling about that” (male*, *age 57*, *solo practice)*.

#### Collaboration and trust: Physiotherapists’ perspective

Table 5 presents the barriers and facilitators that physiotherapists reported. Physiotherapists started with their own treatment options if a patient visited them. They said to sometimes consult a colleague for their expertise. For most physiotherapists it was evident to consider the consultation of a manual therapist in particular neck pain cases. However, the point in time differed. Physiotherapists’ confidence in their own ability, and their trust in the approach of the manual therapist played a role herein.

Physiotherapists pointed out they were trained to treat neck pain and wanted to do so until they reached the borders of their abilities. They told it could be difficult to outsource the care or “*letting someone go*” to the manual therapist as they hoped their own treatment would be successful. Therefore, “*being vulnerable*” and “*putting aside your arrogance*” were mentioned as important individual competencies. Physiotherapists and manual therapists said they observed the beginning of a shift in the profession and training of physiotherapists whereby they were going to look better and better who needed to be the therapist for a particular patient. A recently graduated physiotherapist explained:

*“I also treat arthrogenic problems sometimes*, *but I think if you have a manual therapist who is educated in it*, *why would you not use it*? *I think that is best for the patient*, *better than you yourself are going to fumble with it” (male*, *age 23*, *multidisciplinary health center with MT)*.

The other approach of the manual therapist was sometimes perceived as an added value for the treatment of the patient. One physiotherapist explained:

*“If someone visits me as physiotherapist*, *then I just see what I can do to help the patient*, *with the techniques I learned*. *If the condition does not improve*, *then I consult a manual therapist because he has other techniques and thus you have a slightly different approach*. *That’s the added value then” (female*, *age 30*, *multidisciplinary health center with MT)*.

Besides trust in manual therapy techniques, physiotherapists’ trust in the work of the particular therapist was important. Being familiar with the approach of the manual therapist and being confident that he or she would treat the patient well, facilitated referral, either inside or outside the practice. The last quoted physiotherapist explained how this insight also helped in communication with the patient:

*“I know that if I send people to [name direct colleague manual therapist]*, *the chance that they will be cracked is quite small*. *She first tries all sorts of other things*. *So I inform people about that and that can ease their fear”*.

Lack of trust in the work of the person who performs the treatment impeded referral. This was illustrated by a physiotherapist who told he had referred a few patients to a manual therapist in the neighborhood of his small practice for spinal manipulation, but stopped doing so after bad experiences (e.g. negative stories from the patients and no feedback from the therapist) and now referred such patients to a chiropractor with whom he did have good experiences.

### Organizational context

#### The availability of manual therapists

The availability of manual therapists played a role among the different stakeholders. GPs preferred to refer patients to therapists within their local network with whom they have “*short lines*” (e.g. with the therapist in the same health care center), while sometimes a manual therapist was not or only limited available. GPs also said to be reserved in referring specifically for manual therapy in order to prevent a long waiting time for the patient.

However, some patients said to be willing to bridge the time until a manual therapist was available (e.g. more than a week). One patient interviewed with a manual therapy referral from his GP actually told that he was not willing to wait until a manual therapist was available and decided to be treated by a physiotherapist who could start his treatment on short notice.

Physiotherapists also mentioned the limited capacity and the full agenda of the manual therapists they worked with as a reason to be reserved in consulting the manual therapist during treatment. Others were impeded because of a lack of availability in their practice. For example, one physiotherapist told that she sometimes did advise to visit the manual therapist, but some female patients only wanted to be treated by a woman, while there was no female manual therapist working in the practice.

#### Organization within the physiotherapy practice

The way a practice was organized influenced whether and at what time during the treatment patients were treated by a manual therapist. Practices with both physiotherapists and manual therapists differed in their vision on and policies for treatment and collaboration between therapists. Some therapists told they worked in a practice that used the ‘specialist model’, whereby patients were treated by the therapist who was specialized in treating particular complaints. Hence, patients with neck complaints were usually directed to and treated by a manual therapist.

Most therapists said that within their practice, in principle, each therapist treated neck complaints, since all therapists were competent to treat it. If patients applied at the practice without a referral and without a specific preference for a particular therapist, patients were scheduled according to availability of the therapists. A colleague was consulted if treatment stagnated or when the therapist was at the limits of his abilities. A good culture of collaboration between therapists ensured this worked properly.

If forwarding patients towards a manual therapist meant loss of income, this formed a barrier. For example, because there was no manual therapist working in the practice or because the therapist was paid per consult and his agenda would not be filled by another patient. A physiotherapist who worked solo explained:

*“Ultimately*, *we have chosen for this profession for two reasons*. *One*, *you want to help the patient as best as possible*. *And two*, *you have to think about your own livelihood*. *[…] Look*, *it is never pleasant to refer a patient*, *[…] but I am honest to myself in such a case*. *And then I say*, *yes*, *it is better there*, *and there will be more new patients*. *And I can say it here because the practice is going very well*. *I can imagine that if it is going less well*, *I will be more inclined to retain my patients*. *That’s just the way it is*. *You cannot refer everyone*, *that is just not possible” (male*, *age 24*, *solo practice)*.

#### Regulations and alignment between GPs and therapists

Finally, regulations about treatment after referral influenced the patient flow. Therapists pointed out that if a patient entered their practice with a referral for physiotherapy, formally a practice cannot deviate from the GPs referral and start manual therapy treatment. Therapists acted differently in those cases. Some provided feedback to the GP and requested a manual therapy referral, while others did not or were less tended to discuss it with the GP, as a manual therapist said: *“If the GP says*: *‘you have to do this or that’*, *who are we to say*: *‘no*, *we are not going to do it’”*, and also added: “*the GPs won’t appreciate it*, *if I would do that for each patient” (male*, *age 29*, *PT/MT group practice)*. Manual therapists told they were careful in providing GP feedback about their referral behavior, since they wanted to maintain a good relationship with the GP. Actually, most GPs told they never received such feedback about their referrals, but said to be open for receiving feedback about their referral behavior.

Some GPs already anticipated on these regulations on treatment after referral. For example, a GP told she always wrote both options on the referral letter (‘physiotherapy/manual therapy’) for patients with neck pain so that manual therapy could be started immediately if considered necessary by the therapists. Some GPs even told they did not write a referral letter at all since physiotherapy as well as manual therapy were directly accessible nowadays, but therapists pointed out this was not financially beneficial for the patient as this costs an extra screening consult.

## Discussion

In this study three main aspects were identified, which comprise factors that frequently could be barriers as well as facilitators for referral to manual therapy in neck pain management by patients, GPs and physiotherapists. First, the view on manual therapy for neck pain was for all stakeholders an important aspect. The overlap between physiotherapy and manual therapy impeded referral to the latter; GPs, patients and some physiotherapists found it difficult to make a clear distinction between the two therapies, and therefore generally chose the more familiar option, physiotherapy. Individual perceptions such as knowledge and beliefs about manual therapy for neck pain either impeded or facilitated referral. For example, the perception of a manual therapist as a physiotherapist with expertise or extra education in the neck region was a facilitator. However, fear for complications associated with cervical manipulation was an important barrier for patients as well as GPs. Secondly, collaboration and trust were important concepts. The trust in a particular practitioner was important for GPs, physiotherapist and patients, and existing relations between patients and professionals formed referral behavior. For GPs and physiotherapists it was also important whether they perceived it was part of their professional role to refer for manual therapy. Finally, the organizational context played an important role, wherein the availability of manual therapy could be a barrier for all stakeholders.

The identified main aspects like the view on manual therapy, collaboration and trust between stakeholders, and the organizational context, show that there is a complex and interactive social process underlying referral for manual therapy in neck pain management. The results of the study suggest that the existing barriers and facilitators are quite dependent on the local context. For example, related to the available expertise and the existing collaboration between the professionals involved.

To our knowledge, no previous study has focused on barriers and facilitators for manual therapy referral in neck pain management. The barriers and facilitators identified largely correspond with determinants of innovations in patient care described by Fleuren et al. [18], [22].

Although patients could visit the manual therapist directly, the GP as well as the physiotherapist were often seen as the first port of call when patients requested care for their neck pain. Patients requested care when self-management measures were no longer effective, similar to findings of Scherer et al. [23]. Contrary to the findings of Scherer et al. [23], most patients presenting to the GP seemed to be interested in professional medical counsel and did not only wanted to have their self-diagnosed treatment needs fulfilled. However, the possibility of direct access to the physiotherapist and manual therapist in the Netherlands could explain this different ratio. Further, most of the patients who visited a physiotherapy practice seemed to be receptive to advice of the receptionist or their practitioner about which therapist to see. Proper instruction of the receptionist may increase the number of patients visiting a manual therapist. Patients’ lack of knowledge, beliefs and fear of complications were important barriers to visit a manual therapist for their neck complaints. These barriers can probably be reduced by adequate information and counseling of patients, as has shown to be effective in diabetes care [24].

GPs stressed the importance of taking into account the experiences and preferences of the patient in tuning their advice (including decisions about physiotherapy), which is also found in other studies [16], [25]. The GPs’ decision which therapy was indicated was delegated to the therapists as a routine as GPs thought this to be beyond their competency, resembling the findings of Schers et al. [16]. Another barrier influencing GPs referral behavior was that GPs consider manual therapy as an inactive and somatic treatment that does not match the approach to pay attention to psychosocial factors and active self-management for patients. This in line with a German study that found that GPs mainly focused on an active lifestyle and psychological aspects in managing patients with neck pain [25]. Our findings suggest that to improve GPs’ consideration of manual therapy for patients with neck pain, it is necessary to take into account their role orientation and view on the nature of manual therapy for neck pain, next to their lack of knowledge about the indication for manual therapy for neck pain.

At the professional level, similar factors were found for physiotherapists and GPs, such as professional role orientation (e.g. starting with their own treatment options). However, for physiotherapists more barriers and facilitators were found in their organizational context. These factors showed resemblance with findings of Perreault and colleagues [26], who investigated factors influencing physiotherapists’ inter-professional practices, and found factors such as the organizations’ vison, provider shortage, rules of funding agencies, and hierarchy between professions. To significantly change physiotherapists’ routine in their decisions about consulting a manual therapist in neck pain management, it seems to be necessary to intervene on an organizational level as well. For example, by addressing the patient flow within a physiotherapy practice, but also by making agreements between therapists and GPs about streamlining referrals.

Our study investigated referral to manual therapy from multiple perspectives and disciplines. With regard to professional collaboration and consultation we found factors such as inter-professional trust and the local alignment of roles of the professionals involved. These concepts show overlap with the ‘soft’ factors that can influence the onset and maintenance of successful collaboration in primary care described by Ouwens, Bosch and Wensing [27]. It is important to pay attention to these ‘soft’ factors, next to the ‘hard’ factors, such as financial aspects and regulations.

Possible ways to change GP and physiotherapist practice include strategies that target both the professional and organizational level. For example, by organizing joint educational and interactive sessions for GPs, physiotherapists and manual therapists, and taking steps to gain more insight in the other domain and in each other’s role orientation, and align professional roles in neck pain management. Interactive group meetings and interdisciplinary training have shown positive effects on professional health care, and reinforcing attitudinal and behavioral changes [28], [29]. Since inter-professional and organizational aspects play a prominent role (e.g. regarding ‘short lines’), a local approach, whereby professionals interact with the professionals in their own working environment, appears worthy of further consideration.

The findings of this study provide hints for improving neck pain management in primary care. Context-specific patient-, professional- and organizational-related factors would need to be taken into account and acted upon. A challenging task for further research is the development and evaluation of multifaceted implementation strategies that address the barriers and facilitators identified in this study.

### Strengths and limitations of this study

The particular strength of this study is the use of triangulation of perspectives (i.e. from patients, GPs, physiotherapists, manual therapists and policy makers) and methods (i.e. data collection via interviews and focus groups). Recall bias was minimized by closely relating interviews with patients and practitioners to a recent consultation for neck pain. By complementing the interviews with non-patient specific reflections on the topic, a broader insight was obtained. Moreover, the factors identified in the interviews were assayed in the focus groups.

The diversity of views, motives and behavior may indicate that sample selection was adequate.

Participants’ affinity with neck pain management and manual therapy varied within the stakeholder groups. For example, most GPs and some physiotherapists were not particularly interested in neck pain. Moreover, interview participants did not know that during the interview the focus would be on manual therapy for neck pain.

Practitioners and patients may have given inadequate reports of their behavior, views and motives, but by connecting the interviews of practitioners with those of their patients we tried to avoid this bias as much as possible.

Based on the current data, we have no insight in the frequency and relative weight of the identified factors. However, the methods used gave us insight in important aspects and the room for improvement at the level of the patient, the professional and the organization in the Dutch context.

## Conclusions

This study provides new insights into the factors that facilitate or impede referral to manual therapy for treatment of neck pain. The understanding gained in this study informs the development and implementation of appropriate strategies to optimize referral rates and the use of manual therapy treatment in the primary care management of neck pain. Since barriers and facilitators were found especially in individual perceptions on manual therapy for neck pain, the interaction between the multiple stakeholders involved (patients, GPs and therapists), and the organizational context (e.g. availability of manual therapy), multifaceted implementation strategies that focus on these different aspects seem to be likely to facilitate change in this setting.
